# Using acellular porcine dermal matrix (XCM Biologic® Tissue Matrix) to repair a giant omphalocele: A case report

**DOI:** 10.1097/MD.0000000000033016

**Published:** 2023-02-17

**Authors:** Joo Yeon Park, Jae Hee Chung

**Affiliations:** a Department of Surgery, College of Medicine, The Catholic University of Korea, Seoul, Korea; b Department of Surgery, Seoul St. Mary’s Hospital, College of Medicine, The Catholic University of Korea, Seoul, Republic of Korea.

**Keywords:** closure failure, giant omphalocele, liver herniation, XCM biologic tissue matrix

## Abstract

An omphalocele is an abdominal wall birth defect, and a giant omphalocele (GO) is defined as an omphalocele having a diameter >5 cm or containing a herniated liver. GOs are usually treated in stages and in this case, during the silo reduction, dehiscence occurred at the suture site of the axis ring and skin edge, which was repaired using synthetic absorbable mesh.

**Patient concerns:**

A girl infant was born at 36 weeks with a GO of 8 cm diameter, and herniated multiple organs such as the small bowel, cecum, appendix, and the entire liver. Even after the staged repair technique for the GO silo, wound dehiscence between the ring of the silo and the edge of the skin occurred and gradual reduction failed.

**Diagnosis:**

A GO of 8 cm diameter, which was found during prenatal ultrasonography.

**Interventions:**

Revision was performed to repair the defect. The small bowel and liver were still prolapsed, and there were severe adhesions. After adhesiolysis, the muscle layer of the abdominal wall was repaired using the tissue matrix, but the skin could not be repaired. After the second operation, the defect wound was dressed as sterilely as possible.

**Outcomes:**

The abdominal wall defect was repaired completely; there were no residual complications.

**Lessons:**

Repair of GOs using an acellular porcine dermal matrix can be considered a viable treatment option.

## 1. Introduction

Omphalocele is a congenital anomaly characterized by a midline defect in the anterior abdominal wall with herniation of abdominal contents into a membranous sac.^[1]^ The ultimate goal of surgical intervention in omphalocele is the achievement of fascial and skin coverage, and the avoidance of a physicologically intolerable increase in intra-abdominal pressure.^[2]^

A staged repair technique for giant omphalocele (GO) silo, attached to the fascial margins as a technique for achieving gradual visceral reduction and delayed primary closure of a GO, can be used.^[2]^ However, if the skin opens due to an increase in intra-abdominal pressure during the housing, a reoperation is required to cover it, during which synthetic materials are used. An XCM Biologic® tissue (West Chester, PA) matrix preserves the natural fibrous architecture that provides a scaffold for cell ingrowth and proliferation while allowing revascularization and tissue regeneration. In this case, we attempted coverage using an acellular biologic tissue matrix.

## 2. Case report

A girl infant was born at 36 weeks with a GO of 8 cm diameter, which was found during prenatal ultrasonography. The baby had intrauterine growth retardation and was admitted to the intensive care unit after intubation.

**Figure 1.**
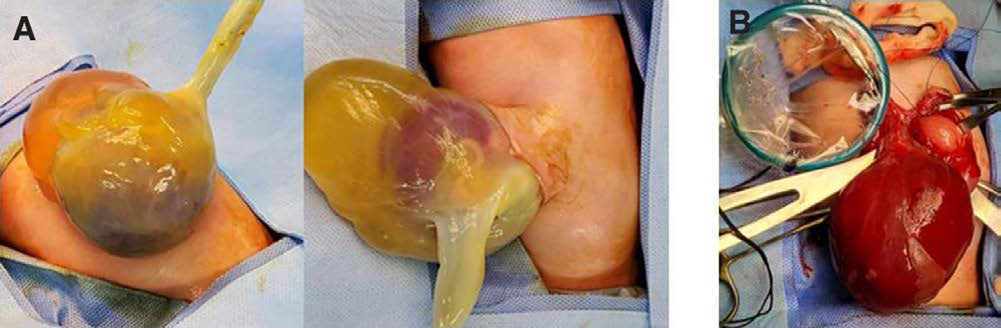
(A) Large omphalocele having prolapse of multiple organs such as small bowel, cecum, appendix, and whole liver. (B) Closure of large omphalocele; housing with Alexis wound retractor.

**Figure 2.**
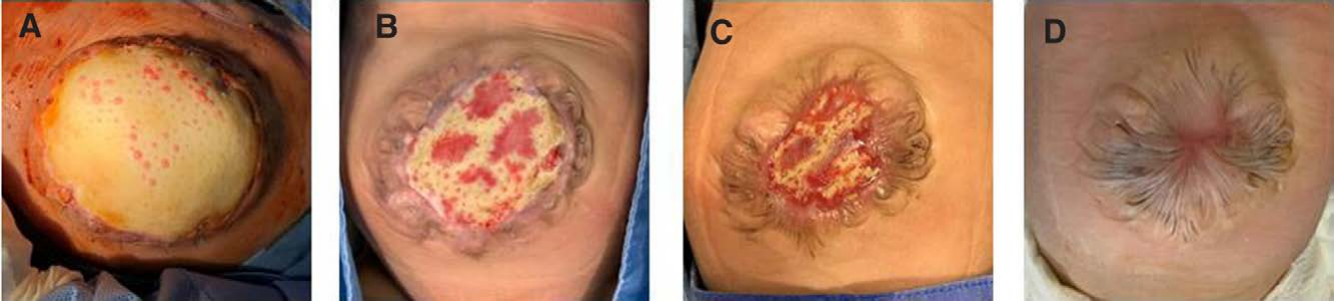
Repair of omphalocele using XCM Biologic® Tissue Matrix after (A) 1 month, (B) 3 months, (C) 6 months, and (D) 12 months – complete epithelialization of omphalocele.

The GO had herniated multiple organs such as the small bowel, cecum, appendix, and the entire liver (Fig. 1). The omphalocele sac was 10 × 6.4 cm in size with a narrow, 3 × 2.5 cm neck. The day after birth, a midline skin incision was made above and below the omphalocele neck, and the liver was separated from the sac (Fig. 1). Using an Alexis wound retractor (Rancho Santa Margarita, CA), a silo was constructed with multiple sutures between the ring of the retractor and the margin of the skin. Daily dressing and gradual reduction of the contents of the silo were performed. On the postoperative 21st day, when wound dehiscence between the ring of the silo and the edge of the skin occurred and gradual reduction failed, revision was performed to repair the defect. The small bowel and liver were still prolapsed, and there were severe adhesions. After adhesiolysis, the muscle layer of the abdominal wall was repaired using the tissue matrix, but the skin could not be repaired (Fig. 2). After the second operation, the defect wound was dressed as sterilely as possible. One month later, the cells started ingrowing through the pores of the mesh and subsequently, fused with each other. Above the cell ingrowth, epithelialization occurred from the skin edge. Twelve months later, the abdominal wall defect was repaired completely; there were no residual complications (Fig. 2).

**Figure 3.**
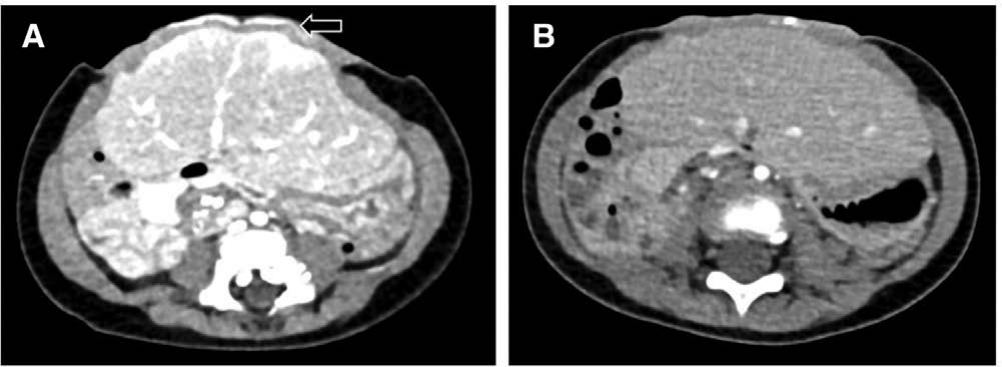
(A) Four months after GO repair, the white line represents the mesh (arrow) of the abdominal wall defect. (B) After 30 months, the mesh above the liver was displaced by muscle tissue. GO = giant omphalocele.

An abdominal pelvis computed tomography was performed during follow-ups 4 months after GO repair and 30 months later (Fig. 3). We checked the Hounsfield unit (HU) value, which expresses computed tomography numbers of the mesh and muscle in a standardized and convenient form. Figure 3A shows an anterior abdominal wall defect approximately 6 cm in size, which is partially closed with the biologic mesh. Compared with the mean HU value of 83 of the rectus abdominis muscle, the mean HU value of the mesh portion of the abdominal defect is measured as 126. The average HU value of the mesh part in Figure 3B is 92, indicating that the mesh has been displaced by muscle tissue.

## 3. Discussion

Omphalocele is a midline defect in the anterior abdominal wall that results in the herniation of abdominal contents into a membrane-covered sac.^[1]^ Rupture of the sac increases the risk of infection and can lead to intestinal or hepatic trauma, while also limiting the options for delayed closure strategies.^[1]^ The ultimate goal of surgical intervention in omphaloceles is the achievement of fascial and skin coverage, and the avoidance of a physiologically intolerable increase in intraabdominal pressure^.[2]^ The degree of viscero-abdominal disproportion often makes it difficult to close the defect in a single stage without causing hemodynamic or respiratory compromise.^[3]^ In 1967, Schuster described the use of a silicone plastic “silo” to provide staged reduction for children with GOs.^[4]^ The viscera are gradually reduced via daily silo until delayed closure is achieved. At the time of closure, the amnion can be simply inverted into the abdomen, which is particularly useful if a mesh is required to repair the fascia, or it can be excised if that facilitates delayed primary fascial closure.^[5]^ The decision to use mesh should be taken based on the size and location of the residual fascial defect. If the defect is small and central, and particularly, if there is residual covering amnion that would prevent acute herniation of the bowel through the defect, it may be best to avoid the mesh altogether and accept a small ventral hernia that can be closed later.^[5]^ The time to resumption of enteral feeding, however, may be shorter with primary closure, although this finding may be affected by the size of the omphalocele and comorbidities.^[4–7]^

In this case, during the silo reduction, dehiscence occurred at the suture site of the Axis ring and skin edge, which was repaired using synthetic absorbable mesh. XCM Biologic® Tissue Matrix is a sterile, acellular, non-cross-linked, 3D matrix derived from the porcine dermis. It can be used as a strong biologic implant with the properties needed to facilitate soft tissue healing. Once the tissue matrix is implanted, it provides a natural fibrous architecture acting as a scaffold for cell ingrowth and proliferation and allows revascularization and tissue regeneration. This mesh is indicated for use in general surgical procedures for hernia repair, and defects of the thoracic wall.^[8–11]^ An acellular dermis is a network of collagen and elastin fibers, proteoglycans, and vascular channels that can serve as a framework for the ingrowth of host connective tissue cells.^[12]^

In this study, when the silastic silo closure failed, we used an acellular porcine dermal matrix, and there were no complications due to the mesh. Therefore, the repair of GOs using an acellular porcine dermal matrix can be considered a viable treatment option.

## Author contributions

**Conceptualization:** Jae Hee Chung.

**Formal analysis:** Jae Hee Chung.

**Investigation:** Jae Hee Chung.

**Project administration:** Jae Hee Chung.

**Resources:** Jae Hee Chung.

**Supervision:** Jae Hee Chung.

**Visualization:** Jae Hee Chung.

**Writing – original draft:** Joo Yeon Park.

**Writing – review & editing:** Jae Hee Chung, Joo Yeon Park.
